# Treatment-Emergent Resistance to Asciminib in Chronic Myeloid Leukemia Patients Due to Myristoyl-Binding Pocket-Mutant of *BCR::ABL1/A337V* Can Be Effectively Overcome with Dasatinib Treatment

**DOI:** 10.3390/curroncol32020097

**Published:** 2025-02-09

**Authors:** Peter Batar, Gabriella Mezei, Arpad Illes

**Affiliations:** 1 Division of Hematology, Institute of Internal Medicine, Clinical Center, University of Debrecen, H-4002 Debrecen, Hungary; gmezei@med.unideb.hu (G.M.); illes.arpad@med.unideb.hu (A.I.); 2 Department of Hematology, Faculty of Medicine, Doctoral School of Clinical Sciences, University of Debrecen, H-4002 Debrecen, Hungary

**Keywords:** chronic myeloid leukemia, tyrosine kinase inhibitor, allosteric inhibitor, TKI resistance, single nucleotide variant

## Abstract

Despite the groundbreaking success of tyrosine kinase inhibitor therapy, the management of chronic myeloid leukemia patients is often impaired by resistance due to specific point mutations in the *BCR::ABL1* oncogene. Upon classical ATP-competitive inhibitor treatment, these single nucleotide variants occur in the tyrosine kinase domain of ABL1. The novel allosteric BCR::ABL1 inhibitor asciminib was developed to treat CML patients alone or in combination to overcome or potentially prevent these treatment-emergent TKD mutations. Here, we present a case of a patient undergoing first-line asciminib therapy, and subsequently develop a specific *BCR::ABL1/A337V* point mutation, which resulted in asciminib resistance. Switching to second-line dasatinib treatment successfully overcame asciminib resistance and helped to achieve a deep molecular response. In case of treatment failures caused by single asciminib-specific point mutations, dasatinib therapy is a feasible choice.

## 1. Introduction

While the currently available tyrosine kinase inhibitors (TKIs) for the chronic phase of Philadelphia chromosome-positive chronic myeloid leukemia (CML) enable a highly effective treatment control of the disease, *BCR::ABL1* single nucleotide variants (BSNVs) pose a real challenge for a great proportion of patients. These specific point mutations can decrease TKI-binding affinity to the ATP-binding site of BCR::ABL1, resulting in partial or complete TKI resistance. Second- and third-generation TKIs have been developed to alleviate imatinib resistance. However, the more potent TKI inhibition resulted in a broad range of additional side effects, and secondary resistance to TKI treatment of 20–30% is still reported [1,2].

Tyrosine kinase domain (TKD) mutations of ABL1, attributed to the genetic instability of the disease, are the most common reason for TKI resistance [3,4]. In the registration trial, 7.5% and 4.8% treatment failure were reported due to imatinib resistance in the second and third year of therapy, respectively [5]. On the contrary, rapid development (<12 months) of resistant clones was associated with advanced-phase disease. These observations suggest that specific resistant clones may be present at the time of diagnosis and selected for TKI therapy as opposed to being induced by TKI therapy itself [6]. Classical ATP-competitive inhibitors bind to the ATP-binding site of the tyrosine kinase domain of ABL1. Point mutations reported upon ATP-competitive inhibitor treatment are mostly located in the TKD region of ABL1. The novel BCR::ABL1 inhibitor asciminib was developed to overcome or potentially prevent treatment-emergent TKD point mutations when used alone or in combination with other ATP-binding catalytic-site TKIs [7].

## 2. Case Report

**Figure 1:**
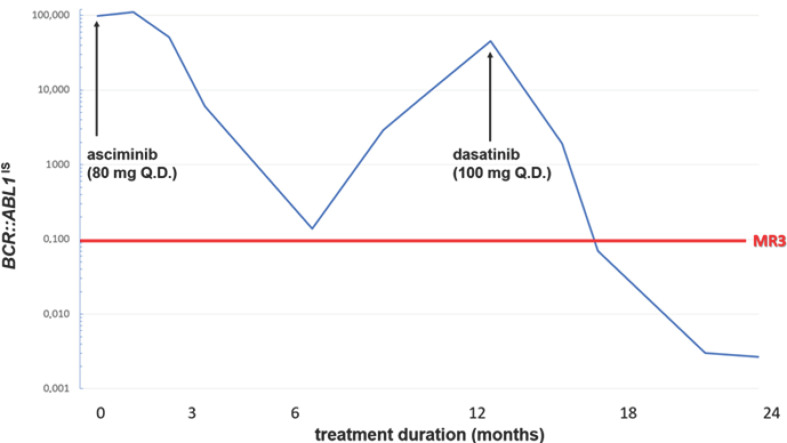
BCR::ABL1IS profile of the patient according to asciminib and dasatinib treatments.

Here, we present a case of a 66-year-old male patient diagnosed with a chronic phase of Philadelphia chromosome-positive CML. Leukocytosis (WBC: 336 × 10^9^/L, left shifted blood work, 5% myeloblast), anemia (HGB: 90 g/L), thrombocytosis (PLT: 927 × 10^9^/L), and splenomegaly (10 cm below left costal margin) were presented at diagnosis. Cytogenetic examination identified the presence of t (9;22) (q34;q11.2) with no additional chromosomal abnormality (ACA). A typical p210 *BCR::ABL1* e13a2 (b2a2) variant was identified by reverse transcriptase polymerase chain reaction. Intermediate risk was verified by ELTS score (1.7601), and hydroxy-urea treatment (1500 mg q.d.) was initiated. Once the diagnosis was confirmed, the patient consented to participate in a clinical trial (CABL001J12301) investigating first-line asciminib treatment vs. investigator-selected TKI therapy (a choice of imatinib, bosutinib, dasatinib, or nilotinib) and was randomized into the asciminib arm of the study. Asciminib (80 mg q.d.) was well tolerated and complete cytogenetic and excellent molecular responses (*BCR::ABL1*^IS^: 0.139%,) were achieved by 6th month. Despite continuous asciminib treatment, losses of hematologic, cytogenetic, and molecular responses were confirmed at 12th month. No compliance issue was raised. Cytogenetic examination showed no ACA. Mutation analysis of the *BCR::ABL1* gene has revealed a specific *A337V* mutation of *BCR::ABL1* known to result in asciminib resistance. The allele frequency of the *A337V* mutation was 95.97%. The patient was withdrawn from the clinical trial and dasatinib (100 mg q.d.) was started. At the time of initiation of dasatinib treatment leukocytosis (WBC: 18.24/10^9^/L), left-shifted blood work (without the presence of blast cells) and thrombocytosis (PLT: 1142 × 10^9^/L) were detected. Complete hematologic response was achieved after 5 weeks of dasatinib treatment (WBC 4.9 × 10^9^/L, PLT 230 × 10^9^/L). Subsequently, complete cytogenetic and major molecular response (MR3, BCR::ABL1^IS^: 0.070%) at 3 months and ultimately deep molecular response (MR4.5, BCR::ABL1^IS^: 0.002%) at 12 months were reached (Figure 1). No side effects of any kind were observed during dasatinib treatment. Due to the deep molecular response, *BCR::ABL1* transcripts were below the limits of detection. Thus, a valid assessment of the *A337V* variant allele frequency was technically not possible. According to our knowledge, this is the first clinical evidence of successful dasatinib treatment after failure of asciminib therapy due to the specific myristoyl-binding pocket-mutant of *BCR::ABL1/A337V*.

## 3. Discussion

Treatment failure due to BSNVs results in a high-risk disease in patients receiving TKI therapy for CML [1]. While classical competitive BCR::ABL1 inhibitors (imatinib, dasatinib, nilotinib, bosutinib, ponatinib) specifically target the ATP-binding catalytic site on the ABL1 domain, asciminib uniquely binds to the myristoyl pocket, which is located outside of the ATP-binding site and triggers specific sterical changes of the protein, converting it to an inactive kinase conformation. Although both ATP-binding site inhibitors and asciminib are all known to induce very similar cellular activities, they exert the development of distinct patterns of *BCR::ABL1* resistance point mutations (ATP-binding pocket vs. myristoyl pocket) and different side effect profiles [7]. Since the ATP-binding site is highly conserved across the human kinome of more than 500 kinases and particularly within the tyrosine kinase family, the off-target activities of TKI treatment often lead to a broad range of adverse effects, resulting in TKI intolerance [8,9]. However, the myristoyl pocket-binding feature of asciminib can reduce these off-target side effects because of the limited number of kinases containing myristoyl-binding sites. In addition, the safety profile of asciminib and its differentiated mechanism as an allosteric inhibitor allows asciminib to be used in combination with other TKIs, providing dual inhibition of the activated BCR::ABL1 in case of compound mutations [7,10].

Among 141 patients reported to have been treated with asciminib in a phase I trial (relapsed, refractory, or intolerant of two or more TKIs), 10 newly emerging mutations were detected in four patients [11]. In a phase III trial of third or subsequent lines of TKI therapy, 157 patients were receiving asciminib [12]. In the asciminib arm, 17 patients were identified with *BCR::ABL1* mutations. Only one patient had a mutation in the myristoyl pocket region, while 16 patients had various ATP-binding site mutations. The percentage of patients with newly developing mutations at the end of treatment was 25.6% on the asciminib and 6.7% on the bosutinib arm [12]. In a phase III trial of first-line asciminib treatment vs. investigator-selected TKIs, the frequency of baseline *BCR::ABL1* mutations assessed using next-generation sequencing (NGS) was low in both the asciminib and investigator-selected TKI groups [13]. Newly emerging mutations during TKI treatment were identified in eight patients (4.0%) receiving asciminib and in four patients (2.0%) receiving investigator-selected TKIs. Mutations observed with asciminib were predominantly in or near the myristoyl pocket. Of the 14 patients who discontinued treatment of asciminib due to unsatisfactory therapeutic effect or disease progression, 8 had newly emerging *BCR::ABL1* mutations [13].

The specific mutant of *BCR::ABL1/A337V* hinders the inhibitory activity of asciminib [13,14]. There are several different BSNVs that are known to result in asciminib resistance (V468F, P465S, A337V, P223S) [7]. In the case of the *A337V* mutation, the change of alanine at position 337 to a larger side-chain amino acid, like valine, sterically alters the bending of helix-αI. This leads to incomplete docking of the SH2 and SH3 domains to the TKD of ABL1, subsequently resulting in an inactive state of the BCR::ABL1 protein. Impairment of the assembly of this inactive state could explain the significant reduction in the activity of asciminib towards this mutant [15].

Sanger sequencing (SS) has traditionally been used to detect *BCR::ABL1* TKD mutations. Using NGS technique has revealed substantially more sensitivity for low-level BSNVs. In a study that included patients with up to five lines of TKI therapy and developed suboptimal responses, 30–40% of patients were reported to harbor low-level TKI-resistant mutations [16]. Low-level mutations were defined by the authors as frequencies between 3–20% and the definition was based on the difference between lower limits of detection by SS and NGS. These low-level mutations eventually could be selected unless TKI treatment was changed [16].

Sequential monitoring of *BCR::ABL1* point mutations by NGS in patients treated with asciminib demonstrated clonal expansion of BSNV-harboring clones, which were associated with asciminib resistance. These data imply that asciminib exerts selective pressure on some BSNV-harboring populations in vivo, some of which may only respond when TKI therapy is modified accordingly [17].

Compound mutations of *BCR::ABL1* lead to resistance against multiple TKIs, posing an additional challenge in the treatment of CML. These compound mutants can be effectively eliminated using a combination of the ATP-site-specific inhibitors nilotinib or ponatinib with the allosteric inhibitor asciminib [7,10]. This approach is beneficial because asciminib has a resistance profile that differs from the ATP-binding catalytic-site inhibitors targeting BCR::ABL1 [7].

The restoration of BCR::ABL1 signaling as a result of point mutations in or close to the myristoyl pocket is one of the mechanisms of acquired resistance to asciminib therapy [13,18]. Classical ATP-binding site TKIs were able to counteract myristoyl-binding site mutation-based asciminib resistance, according to in vitro data from cell-based studies [18]. Dasatinib was discovered to be the most effective classical TKI in inhibiting these asciminib-resistant BCR::ABL1-positive cell lines [18].

## 4. Conclusions

There is a lack of information regarding the management of patients harboring asciminib-specific BSNVs. Treatment options after failure of asciminib therapy in the case of multiple lines of previously applied ATP-binding catalytic-site TKIs are extremely limited (clinical trial, allogenic stem cell transplantation). However, as promising clinical data on the efficacy and safety of asciminib treatment are accumulated, initiation of asciminib treatment in earlier lines of CML therapy is becoming more feasible [13]. Therefore, monitoring for newly emerging point mutations close to the myristoyl-binding sites of BCR::ABL1 during asciminib therapy is extremely important. In the case of a specific BSNV, subsequent TKI treatment should be carefully chosen according to the type of mutation and the resistance profile of the selected TKI.

To our knowledge, there has been no report in clinical practice so far of subsequent dasatinib therapy in case of asciminib treatment failure due to a single *BCR::ABL1/A337V* point mutation. Although dual-targeting treatment (ATP-binding catalytic-site inhibitors plus asciminib) can be a feasible choice for compound mutations, in case of treatment failure due to a single asciminib-specific BSNV, dasatinib therapy is an appropriate choice for this group of high-risk CML patients.

## Data Availability

The data presented in this study are available upon request from the corresponding author due to ethical reasons.
